# Patterns of verbal interaction in newly formed music ensembles

**DOI:** 10.3389/fpsyg.2022.987775

**Published:** 2022-11-01

**Authors:** Nicola Pennill, Renee Timmers

**Affiliations:** Department of Music, The University of Sheffield, Sheffield, United Kingdom

**Keywords:** music groups, verbal behaviour, T-pattern analysis, case study, emergence

## Abstract

Ensemble rehearsal in the European classical music tradition has a relatively homogenised format in which play-through, discussion, and practice of excerpts are employed to establish and agree on performance parameters of notated music. This research analyses patterns in such verbal communication during rehearsals and their development over time. Analysing two newly established ensembles that work over several months to a performance, it investigates the interaction dynamics of two closely collaborating groups and adaptation depending on task demands, familiarity with each other and an upcoming deadline. A case study approach with two groups of five singers allowed in-depth exploration of individual behaviours and contributions; results are reported descriptively and supported by qualitative data. The results highlight changes over time that reflect the development of implicit (faster decisions) interactions from explicit (slower decisions). They show a trajectory of opening up and closing down in terms of interactional flexibility, enabling members to significantly contribute to the group, followed by tightening the interaction to establish stability for performance. These findings and novel employment of T-pattern analysis contribute to the understanding of human group behaviour and interaction patterns leading to expert team performance.

## Introduction

Playing with other musicians in a group (‘ensemble’) setting is a common part of professional practice within the European classical music tradition. For performance preparation, this generally takes the form of one or more rehearsals, through which the ensemble develops a shared approach to performance of the music, and solves problems related to execution, often alongside individual preparation which happens away from the rehearsal setting (Keller, 2014). This working environment encompasses social and musical interactions. Ways of working together are often drawn from customary practice, learned from peers and through specialist music education; and are implicitly rather than explicitly agreed as individual and joint goals are negotiated (Macritchie et al., 2018). Given this emphasis on implicit coordination processes, identifying the underlying organisational mechanisms in music groups can be challenging. The approach adopted in this study is to focus on explicit behaviours, in the form of verbal interactions, investigating ways they evolve over time, and analysing the formation and development of verbal interaction patterns.

Nonverbal communication is increasingly recognised as the primary mode of conveying timing and expressive meaning in musical coordination (Timmers et al., 2022), whilst a combination of both verbal and nonverbal communication modes have been suggested to determine the quality of the musical output (Kokotsaki, 2007). The amount, type, and purpose of verbal interactions in music ensembles is idiosyncratic, and subject to changes over time within and across rehearsals (King and Ginsborg, 2011; Ginsborg and King, 2012). Verbal behaviour plays a key role in clarification and consensus building, and in supporting the development of social relationships, but is not conventionally used in performance. Hence, a core purpose of rehearsal is to establish patterns of interactions, which can then be ‘replayed’ (nonverbally) in the moment during performance (King and Gritten (2017), p. 318). This supports the connection between verbal interactions in rehearsal and performance outcomes, in which patterns of behaviour are embodied, and part of the ‘implicit communication strategy’ of performance which Gilboa and Tal-Shmotkin (2012) describe as,

… an implicit communication strategy to make time-critical decisions … the performance phase^1^ combines anxiety and artistry; performance remains mysterious even to the musicians themselves (Gilboa and Tal-Shmotkin, 2012, p. 34).

Whilst there is some evidence of this shift from verbal to nonverbal communication from previous studies, there have been few longitudinal studies with groups, and none to date to explore the transition from ‘communication’ to ‘interaction’ modes (King and Gritten, 2017). In particular, very little is known about the emergence and development of patterned interactions from initial rehearsal to public performance, which the employment of T-pattern analysis can assist with (Magnusson, 2000). Small groups arise in many contexts within human society. When a consensus or collaboration is required, people often come together in groups to cooperate, or share skills and resources. Over the past 50 years, a number of theoretical perspectives on small group working have come to the fore, of particular relevance to purposive, goal-orientated groups, such as music ensembles working towards a future performance (Poole and Hollingshead, 2004). For our research, we adopt a temporal perspective, in which time acts as context for group development, a resource to be allocated, or as a mediating variable; and which foregrounds process over outcome (Arrow et al., 2004). Within this framing, our focus was on how the groups under consideration systematically changed over time, and the role of patterned interactions in those changes. Prior research in a workplace setting has shown that groups working on projects with deadlines exhibit temporal patterning in their interaction, which are reflected in the internal rhythms and pacing by which they structure and coordinate activities. These patterns include the ‘midpoint transition’, whereby a marked change in behaviour occurs around halfway through a given preparation period (Gersick, 1988; McGrath, 1991), and which can be attributed to a number of causes, including deadline pressure and evolving team dynamics (Seers and Woodruff, 1997). Gersick and Hackman (1990) posit that these patterns are indicative of a groups’ efforts to establish routine, and that patterns persist unless a new focus or challenge arises, such as that which might arise when deadline pressure increases. They further propose that changes in patterns are influenced by the severity and frequency of changes and hypothesised that ‘importation’ of task habits (whereby prior experiences shape a new setting), the creation of unique new patterns, and their evolution, are all factors in how new groups establish themselves and develop over time. These factors may be also subject to further variation in the absence of formal roles or where groups are under greater time pressure or experiencing different task demands (Kelly et al., 1997).

In order to investigate such temporal phenomena and behaviours, a number of researchers have adopted temporal pattern (‘T-pattern’) analysis as part of a mixed-methods approach to reveal self-similarity in sequences of behaviours (Magnusson, 2020), for a review, see Casarrubea et al. (2015). T-pattern analysis can detect the presence, timing, and complexity of patterns of repeated behaviours such as verbal and social interactions and can contribute to a greater understanding of ways that members of a group work together, including turn-taking, brief sequences of verbal behaviours and to identify emergent changes or transitions. Interaction patterns have been studied in a range of settings including small groups of professionals working to deadlines (Ballard et al., 2008), in crisis situations (Stachowski et al., 2009) and in emergency teams (Zijlstra et al., 2012). The THEME^®^ software algorithm (Patternvision Ltd) was chosen as the analysis tool for pattern detection, which in group behaviour research has been used to investigate interactions during sports (Camerino et al., 2012; Pic and Jonsson, 2021), in contemporary dance (Castañer et al., 2009; Torrents et al., 2013; Harrison and Rouse, 2014), as well as in the study of complex interactions in teams during information sharing (Hoogeboom and Wilderom, 2019). These studies provide useful models for our research into groups in the musical context. In addition, T-pattern analysis has been used as a tool in the study of verbal and nonverbal communication; Castañer et al. (2016) investigated ‘paraverbal’ communication in teachers, to identify ways in which kinesic (gesture and posture) and proxemic (use of space) modes were used alongside verbal communication during delivery.

Our study investigated the changes over time in the verbal interactions arising during performance preparation in two small, newly formed music ensembles in a series of rehearsals. The primary aim was to identify T-patterns of interaction to highlight the existence of underlying structures in the real-time behaviour, and the contributions of group members. We had the unique opportunity to gain insights from the early start of an ensemble (first time they came together). This allowed us to follow the changes in interaction, how ensemble members contributed to the progression of the rehearsal process, and how changes of tasks and task context may contribute to changes in T-patterns. Investigating interaction patterns offers insight into working practises amongst highly specialised team members including insight into patterns of distributed and democratic leadership vs. hierarchical or autocratic forms of direction (Bathurst and Ladkin, 2012).

As such the following research questions were addressed:

- What were the observable patterns of verbal interaction between ensemble members during rehearsals?
- How did these develop over time, across rehearsals, in the context of changes in rehearsal context from early encounters to polishing for performance?
- What light do these patterns shed onto the roles of ensemble members at various stages of the rehearsals?

## Materials and methods

The music context was a specialist higher education setting, in which postgraduate students, selected by audition for suitability, were participating in a professional practice programme. This setting enabled tracking behaviours in bi-weekly rehearsals of the groups for 2–3 months from day 1 of their musical interactions.

### Participants

The participants were two vocal groups, each comprising five pre-professional level solo singers, at a United Kingdom university. There were three women and two men in each group. They were allocated to vocal parts as follows:

#### Group 1

Singer A, female – soprano.

Singer B, female – mezzo-soprano.

Singer C, female – alto.

Singer D, male – tenor.

Singer E, male – bass.

#### Group 2

Singer V, female – Soprano.

Singer W, female – Mezzo-Soprano 1.

Singer X, female – Mezzo-Soprano 2.

Singer Y, male – Tenor.

Singer Z, male – Bass.

### Materials

Materials for analysis came from video recordings of rehearsals. Verbal exchanges were transcribed and coded using the Behaviour Analysis (BA) observational instrument (Rackham and Morgan, 1977; Farley et al., 2018; see Table 1). Four main categories were used for analysis, as behaviours were grouped into the categories of ‘Clarifying’ (ensuring a common understanding); ‘Initiating’ (to create ideas and possibilities); ‘Reacting’ (to ensure agreement and resolve disagreement); and ‘Participating’ (which bring in or shut out others or lighten the mood through humour). The first author was trained in the use of this scheme prior to coding, and her coding consistency was checked against a benchmarked standard as part of the training she received and checked by an independent coder.

**Table 1:** Behaviour analysis coding scheme used as observational instrument for categorising verbal behaviours.

Behaviour analysis category	Code	Sub-category
Initiating behaviours	I	Proposing ideas
		Proposing behaviours
		Building ideas
Participating behaviours	P	Bringing in
		Shutting out
		Lightening the mood
Clarifying behaviours	C	Giving task information
		Giving personal information
		Seeking task information
		Seeking personal information
		Checking understanding
Reacting behaviours	R	Supporting ideas
		Supporting people
		Disagreeing
		Defending/attacking

#### Group 1

The group was provided with a video camera (Sony MV1 Music Video recorder). In order to minimise disruption to their normal working processes, members of the group were shown how to use the camera and submit the recordings post-rehearsal. They scheduled and directed their own rehearsals, and they were asked to rehearse and interact as normal. Multiple rehearsals were recorded, and the camera became a customary part of their rehearsal process. Rehearsals were dedicated to trying and refining different repertoire. At the end of the rehearsal series, a selection of this repertoire was performed in front of an audience and panel as part of a formal assessment.

#### Group 2

For the second group, five sessions were pre-arranged by the researchers and recorded in a laboratory setting. Musical materials were provided in the form of two original pieces which were created for the purpose of the study (for details of the scores, see D’Amario et al., 2020b). Neither piece had text, nor were sung to the vowel sound ‘e’. No expressive markings were included – the singers were asked to develop their own expressive interpretation as a goal of rehearsals. In the final session, the group were invited to perform for a recording, which took place within the lab setting. The contrast in the structure of the two pieces was primarily in the texture, whereby Piece 1 was in rhythmic unison (‘homophonic’, literally ‘one voice’), and Piece 2 contained multiple, overlapping melodic lines with differences in rhythms to each other (‘polyphonic’ or ‘many voices’). The participants only had access to the material during the session; no rehearsal on these pieces happened outside the study sessions. However, the singers were regularly working together on other materials, both independently and in coached sessions, in the intervening days and weeks between sessions. A single video camera was set up to record all interactions throughout the session, using a tripod-mounted Sony MV1 Music Video recorder. The camera recording was started at the beginning of the session and left running throughout.

### Procedure

The participants were approached to take part in the study before their first rehearsal took place. Ethical approval was obtained for the study and informed consent for participation arranged in time to organise a first recording session in the first week that the vocal group was formed.

The protocol for each group was as follows:

Group 1 were given an initial briefing and shown how to use the equipment. Subsequently, the group were allowed to proceed as they chose, recording rehearsals whenever possible. From these recordings, selections for further study were made based on time-interval to provide regularly spaced samples. Where group members were obscured by camera angles or more than one member was absent, recordings were not used. Four sessions were selected for analysis related to Weeks 1, 3, 5, and 7, and the first 30 min of each rehearsal was analysed. All five singers were present in weeks 1, 3 and 7; in Week 5 Singer B was absent due to illness.

Group 2 were presented with two set excerpts to rehearse. In the first session the task was explained to the participants, and they were asked to prepare both pieces for a possible future performance, and to create an expressive interpretation. The singers were not aware of the purpose of the study. The same procedure was followed each time: the group performed the excerpt of music, rehearsed it for a short, timed period, and then performed it again. This procedure was repeated for the second musical excerpt. The rehearsal sessions were video-recorded and verbal interactions transcribed and coded as for Group 1. The two pieces were randomised for order in which they were presented (see Table 2). Five sessions were recorded over a 16-week period in week 1, 3, 6, 8, and 16, respectively. Apart from controlling the music that was rehearsed, the duration of rehearsals and the rehearsal venue, the participants were asked to work independently as they normally would, and the researcher left the room. The sessions were timed using a digital timer and after 10 min the researcher returned to the rehearsal room at which point the rehearsal stopped. Each session was approximately 1 h long. All five recording sessions were used in the analysis. Only the rehearsal part of the sessions was analysed. Analyses of tuning and synchronisation in the performances have been reported in earlier publications (D’Amario et al., 2020a,b).

**Table 2:** Singing, talking and number of utterances in sampled period (Groups 1 and 2).

	Rehearsal activity	Week 1	Week 3	Week 5	Week 6	Week 7	Week 8	Week 16
Group 1	Singing (% of time)	33.5	28.9	60.9		53.1		
	Talking (% of time)	66.5	71.1	39.1		46.9		
	Number of utterances (*N*)	179	260	250		196		
Group 2	Singing (% of time)	35.1	42.7		31.5		21.8	45.5
	Talking (% of time)	64.9	57.3		68.5		78.2	55.5
	Number of utterances (*N*)	222	159		248		218	251

### Data preparation

Exchanges during rehearsals were transcribed verbatim to produce time-stamped, line-by-line utterances. The first author and a second, independent rater who was also trained in the BA coding scheme coded the utterances with an agreement score (Cohen’s kappa) of 0.67 at sub-category level, comfortably above the ‘substantial agreement’ boundary of 0.6 as defined by Landis and Koch (1977). There was a higher agreement at category level, which was then used in the final analysis. Occurrences and durations of the whole group singing together were also recorded. Only single codes were assigned. During the transcription process the duration of each utterance was noted. A text file was created with the time-stamped output (in seconds) and coded with the person speaking (‘actor’) and the category of behaviour. In the pattern descriptions each pair of letters represents first the singer(s) (A, B, C, D, E; V, W, X, Y, Z; SOME or ALL), followed by the category of interaction (Clarifying (C), Initiating (P), Reacting (R), or Participating (P)). For example, ‘A, I’ described an event type in which Singer A exhibited Initiating behaviour. Two additional categories were included to support the pattern detection; M (Music-making) and N (no specific category assigned). For this analysis, subcategories were not used.

### Analysis

First, the main characteristics of rehearsals and behaviour were analysed examining frequencies of behaviours. Secondly, interaction patterns were analysed, including developments across the rehearsal period. Comparing frequency and complexity of interaction patterns was used as an indicator for how the rehearsal processes are unfolding; for example, how fast-paced decision-making is. This has implications too for how much implicit (related to faster decisions) versus explicit (slower decisions) communication there is.

The amount of time spent singing and talking in the first 30 min (Group 1) or the 2 × 10 minutes (Group 2) was analysed. Patterned interactions were analysed using THEME^®^ v 6.0 and were based on the distribution of behaviour categories and actors over time. The following search parameters were set [based on guidance in the manual (Magnusson, 2022)]: (a) frequency of occurrence of ≥3; (b) significance level of 0.005 (0.5% probability of critical interval being due to chance); (c) deactivation of fast requirement and selection of free critical interval algorithm in which, if present, at least one critical interval is found; (d) validation of results through randomisation of data on five occasions. These settings were arrived at through a series of validation tests with varying flexibility of statistical margins (*p* < 0.05 and *p* < 0.001), and minimum number of occurrences (2 and 4). This is consistent with previous studies, which utilised similar settings (Zijlstra et al., 2012; Amatria et al., 2017; Hoogeboom and Wilderom, 2019; Pic et al., 2021). Repeated sequences of events were detected from which patterns were inferred. Pattern length (number of events in a pattern), number of levels (an index of complexity based on hierarchical structure of the pattern), number of actor switches (an indicator of turn-taking) were used as the basis for summarising the main behaviours. Occurrences of ‘mono-actor’ patterns, where the pattern involved a single actor (Stachowski et al., 2009) were also recorded, as an indicator of balance of contributions – more mono-actor patterns have been reported in less effective groups, which may suggest less ‘balanced’ interaction between group members. Dyadic (two-person) patterns were also recorded, which can provide an indication of emergence of group member social relationships (Kozlowski et al., 1999).

Other events not appearing in patterns were excluded from the subsequent analysis. Each remaining pattern represents a sequence of events that recurs at least three times within a so-called ‘critical interval’. This interval is different for each data set and defined as follows.

‘If A is an earlier and B a later component of the same recurring T-pattern, then, after an occurrence of A at t, there is an interval (*t* + d1, *t* + d2) (d2 ≥ d1 ≥ d0) that tends to contain at least one occurrence of B more often than would be expected by chance.’ (Magnusson, 2000, pp. 94–95).

From the main patterns extracted the verbal content was compared to the original transcript. From this process, a coded description was created.

## Results

Given the differences in goals and settings for data collection, results are presented for Groups 1 and 2 separately, before discussing emerging shared features.

### Allocation of time talking and singing during rehearsal period

For Group 1, there were frequent brief verbal exchanges in all rehearsals, which were highest in number in Week 3. When examined by duration, the total amount of time spent talking was high in Weeks 1 (66.5%) and 3 (71.1%), and less in Weeks 5 (39.1%) and 7 (46.9%), Conversely, time singing together was greatest in Week 5, which also contained the fewest singing episodes (where a singing episode involved the group singing together a passage, movement, or piece). In Group 2, the total amount of time spent talking ranged from a low of 55.5% in Week 16 up to 78.2% in Week 8. This transition from Weeks 8 to 16 indicates a marked shift from a rehearsal where there is much discussion, to one where changes and ideas are tried out by music-making, ready for the final recording. See Table 2 for summary of singing and talking episodes for Groups 1 and 2.

The different profiles of talking/singing in the two groups may be explained by the different settings and tasks, amongst others – Group 1 was more typical of how a group may work over a series of rehearsals towards an end goal, whilst Group 2 experienced shorter sessions, with a change of task, and shorter-term goals for each session. The task switching activity may have generated further stimulus for discussion, and the number of utterances remained a high proportion of the time. The laboratory setting, time-constraints and provided materials made the rehearsal circumstances a bit artificial, and although different from Group 1, this is not entirely unusual when musicians have only limited time to rehearse musical material.

### Behaviour over time and T-pattern detection

T-pattern results are presented per analysed rehearsal, reflecting the rehearsal context and quality of the verbal exchanges associated with each pattern. Possible interpretations are offered having referred to the original transcript and video for further context, and further possible interpretations made of the ways in which individuals contributed to these patterns. Selected dendrograms from the THEME^®^ analysis are used to illustrate features of the emerging patterns.

### Group 1 emerging interaction patterns

First, the basic pattern data is summarised (Table 3) and differences and themes over time by rehearsal session are explored.

**Table 3:** Group 1: Summary of pattern data by rehearsal.

Week	Length of main pattern (N of events)	Event types in patterns	Duration (secs) mean	Duration (secs) S.D.	Actor switches mean	Actor switches S.D.	Duration of patterned behaviour (% total)
1	4	25	2.82	0.86	1.25	0.84	44
3	7	24	3.21	1.26	0.77	0.72	41
5	15	23	5.91	2.39	1.05	1.15	80
7	6	24	2.50	0.88	0.53	0.74	34

#### Group 1, rehearsal 1 (week 1)

The main pattern identified consisted of the sequence [(ALL,N [A,I C,R]) D,P]: This can be qualitatively described as the group engaging in a shared activity, followed by an interaction between singers A and C, whereby A Initiated an event, and C Reacted. This was followed by singer D Participating.

The pattern occurred three times, and the duration of patterned behaviour was 44% of the 30-min rehearsal time. The number of events (pattern length) was 4. From the transcript these were characterised as light-hearted interactions, triggered by a collective activity (e.g., all referring to a musical score) and ending with a jokey remark. There were no mono-actor patterns detected.

#### Group 1, rehearsal 2 (week 3)

Compared with Week 1, the patterns in Week 3 were longer, and more fragmented into sub patterns. Longer patterns have been associated with the development of implicit coordination modes associated with groups adapting to a task (Uitdewilligen et al., 2018).

The main pattern was: (D,P (((E,C B,C)(D,C E,I))(E,C B,C))) comprising;

Singer D – Participating;

Singers E and B – Clarifying;

Singer D – Clarifying;

Singer E – Initiating;

and Singers E and B – Clarifying.

**Figure 1:**
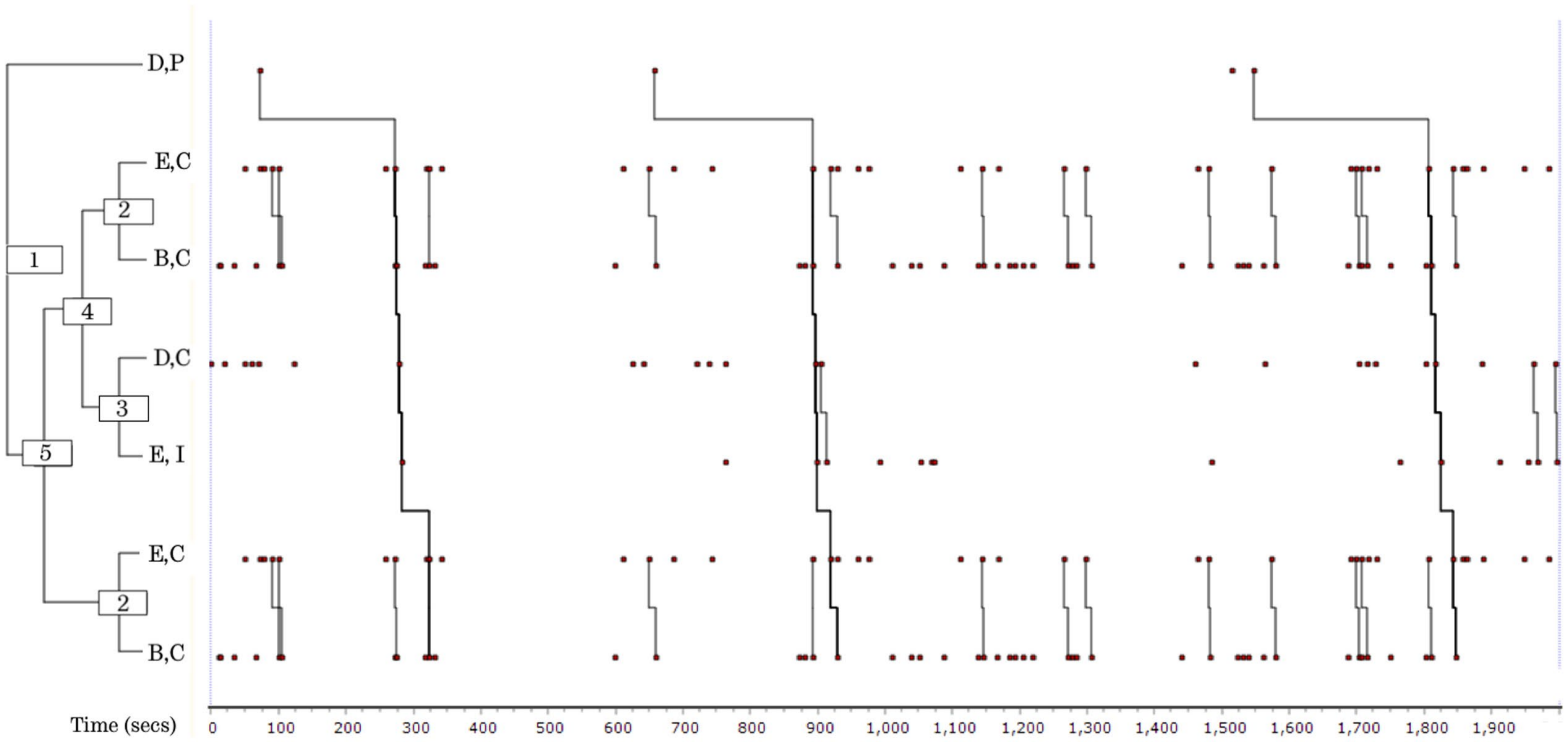
Pattern diagram (dendrogram) output from THEME^®^ analysis showing main patterns and frequent sub patterns (Group 1, Week 3). Three members of the ensemble featured in the patterns; interactions were a mixture of light-hearted and more task-focussed discussions of technical aspects, such as choice of speed, expression, or repertoire. The first event in all long patterns was a humorous contribution from Singer D (1), followed by dyadic interactions between Singers E and B (2), and between D and E (3), which appear in combination (4) and in the main pattern (5).

The pattern occurred three times, and patterned behaviour occupied 41% of the Week 3 rehearsal time. The length of the pattern was 7 events (Figure 1).

#### Group 1, rehearsal 3 (week 5)

**Figure 2:**
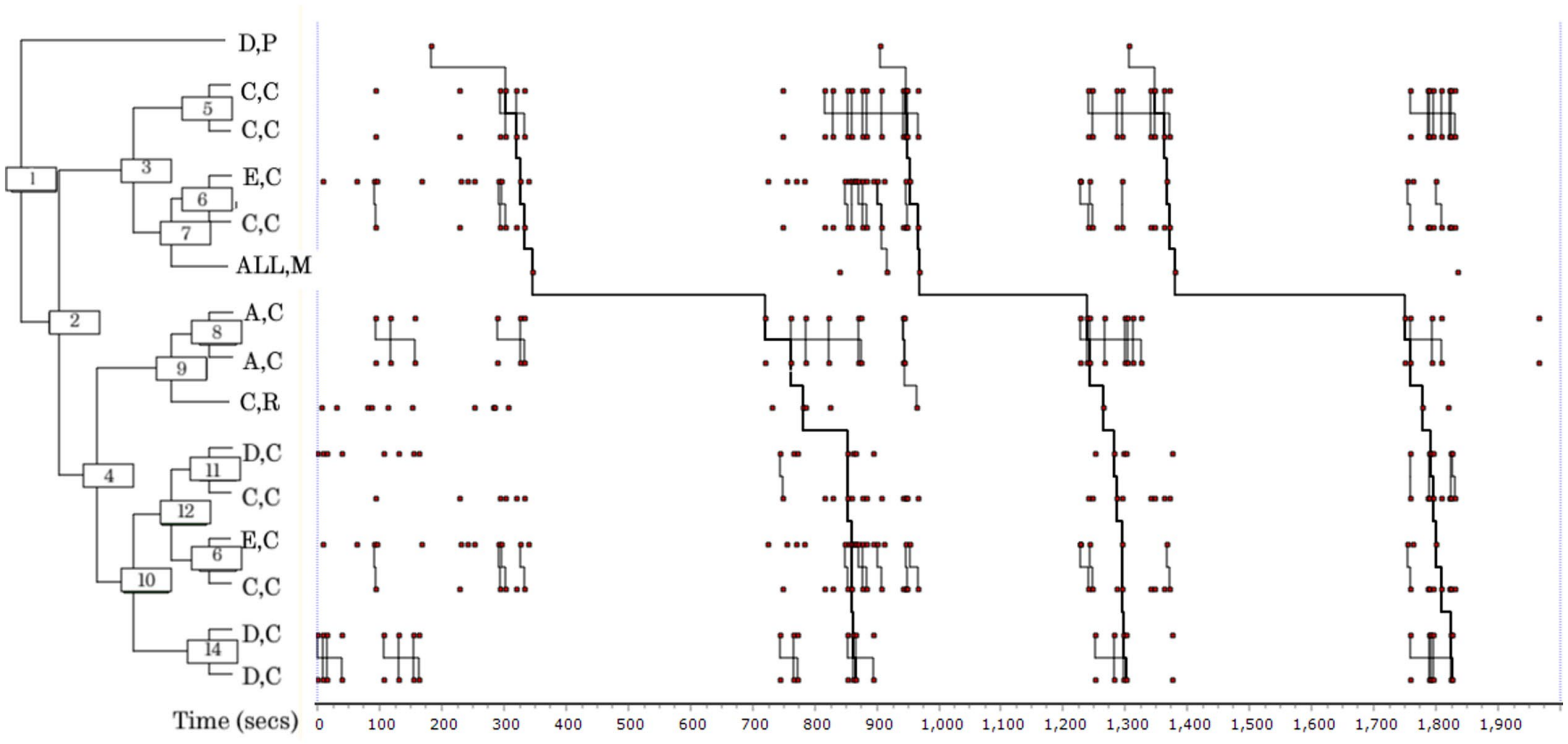
Pattern diagram (dendrogram) output from THEME^®^ analysis showing main patterns and frequent sub patterns (Group 1, Week 5). In this week, patterns precede and follow group singing episodes (All, M). Features include longer patterns and numerous dyadic and mono-actor sub patterns. A light-hearted or humorous contribution from Singer D is followed by mono-actor Clarifying interactions (2, 8, 14) and dyadic exchanges between E and C, and D and C (6, 11). Patterns following singing (4) involved each of the singers engaged in Clarifying, apart from one sub pattern including Reacting (9).

Week 5 was highly patterned. The dendrogram from THEME^®^ (Figure 2) shows short bursts of patterned interaction, prominently featuring Singer C, combined with longer, complex patterns involving all members. The dendrogram also illustrates how the patterns span, and indeed incorporate, an episode of singing. More sub patterns were evident compared to Week 3, in the form of short, dyadic interactions. As with Week 3, Singer D’s Participating behaviour initiates the main long pattern. Otherwise, there are three mono-actor patterns occurring with a high frequency, concerning Singer A (20 occurrences), Singer C (27 occurrences), and Singer D (19 occurrences). This may be due to the absence of Singer B from the rehearsal affecting the dynamics of the interactions between the remaining four members.

The main pattern was (D,P (((C,C C,C) ((E,C C,C) ALL,M)) (((A,C A,C) C,R) (((D,C C,C) (E,C C,C) (D,C D,C))))).

with Singer D – Participating;

Singer C – Clarifying;

Singers E and C – Clarifying;

ALL – Music-making;

Singer A – Clarifying;

Singer C – Reacting;

Singers D and C – Clarifying;

Singers E and C – Clarifying;

and Singer D – Clarifying.

This main pattern occurred three times, occupying 80% of the Week 5 rehearsal time. The length of the pattern was 15 events.

A combination of simple and complex patterns is reflected in the patterns of behaviour which precede and follow a singing episode, in which Singer C has a role in harnessing the ideas to try out, whilst Singer A has a key role in responding to what has been tried:

- Preceding singing: (D,P (((C,C C,C) ((E,C C,C)). This sequence is dominated by Singer C providing suggestions about how to approach the task and seeking to clarify what is required.
- Pattern following singing: ((A,C A,C) C,R) ((D,C C,C) (E,C C,C) (D,C D,C)). Singer A is the first to respond to what has just been tried and Singer C reacts.

#### Group 1, rehearsal 4 (week 7)

In Week 7, there are fewer, simpler patterns. All members except Singer D are involved in the main pattern. There are also fewer sub-exchanges or dyads. Singer E shifts from Clarifying to Initiating mode, and Singer A plays a consistent role in Initiating singing episodes.

The main pattern is (B,C C,C)((E,C E,I)(A,I ALL,M))). This involves:

Singers B and C – Clarifying;

Singer E – Clarifying and Initiating;

Singer A – Initiating;

and ALL – Singing.

This main pattern occurred three times, occupying 34% of the rehearsal period. The length of the pattern was 6 events. There is a high degree of similarity of the qualitative content of the verbal interactions appearing in the patterns consistent with the emergence of a common understanding as the group achieves a greater coherence and consistency. The pattern each time includes checking of pronunciation by Singer A; clarified by Singer C; Singer E gives an opinion on the interpretation or expression; Singer A makes a suggestion relating to the current task, then they all sing a passage or piece.

#### Group 1 – observations across rehearsals

Between Weeks 1, 3 and 5 there was an increase in pattern length and amount of patterned behaviour. In Week 5, patterns were longest with more actor switches (number of different participants in a pattern), as well as the highest duration of patterned behaviour. Week 5 also coincided with the calendar midpoint of the group’s preparation timetable (with a performance in Week 9). This is consistent with previous studies which report groups exhibiting a type of ‘tipping’ point transition around the midpoint, as their impending deadline creates a new sense of urgency and focus (Gersick, 1988, 1989).

In Week 7, after the complex patterns of Week 5, simpler patterns were evident, suggesting that as they approached their performance deadline, the group were achieving greater consistency in their interactions. They need to balance exploratory behaviours with more predictable outcomes.

Some recurring interactions between individuals emerged. Singer E featured more in the patterns from Week 3 and from observation of the group he was vocal and active throughout. Other patterns involved the ‘quieter’ members of the group, and their contribution was accordingly harder to detect using traditional observation methods – for example, the Initiating behaviour of Singer A in Weeks 1 and 7, and the Participating role of Singer D in Weeks 3 and 5. Reviewing the transcripts through the lens offered by the patterns gave a nuanced perspective on how different individual members influenced the overall group dynamics. Table 4 shows how these individual traits were expressed in the patterns. Singers A and E showed Initiating behaviour, often shortly before or after singing. Singer C was the only one to show Reacting behaviour. Singer D often contributed Participating behaviour in the form of humorous remarks which triggered a shift to a new focus.

**Table 4:** Singer behaviour types occurring in patterns, by week.

Behaviour type in pattern	Week 1	Week 3	Week 5	Week 7
Clarifying		B, D, E	A, C, E	B, C, E
Initiating	A	E		A, E
Reacting	C		C	
Participating	D		D	

In summary, the following informal roles were identified:

Singer A: Initiated and follows up after singing episodes.

Singer B: Vocal in early rehearsals due to a technical specialism (language).

Singer C: Often contributed opinions prior to a singing episode.

Singer D: Quiet, but use of humour creates shifts of focus and subsequent interactions.

Singer E: Increasingly active over time from week 3.

Over time, more members of the group were involved in the patterned behaviours from 2 in Week 1 to 4 in Weeks 5 and 7.

### Group 2 emerging interaction patterns

For Group 2, emerging patterns in behaviours for each session were analysed and summarised (Table 5). Additionally, the design allowed for further analyses to explore the ways these patterns and behaviours varied according to the changing tasks.

**Table 5:** Group 2: summary of pattern data by rehearsal.

Week	Length of main pattern (no. of events)	Event types in patterns	Duration (secs) mean	Duration (secs) S.D.	Actor switches mean	Actor switches S.D.	Duration of patterned behaviour (% total)	Total observation time (secs)
1	5	15	2.48	0.77	1.26	0.73	29	1,238
3	3	19	2.30	0.48	1.20	0.63	29	905
5	4	18	2.46	0.63	1.04	0.65	30	1,512
8	8	11	3.29	1.49	1.95	1.16	56	1,234
16	9	18	3.68	1.63	2.04	1.40	47	1,651

#### Group 2, rehearsal 1 (week 1)

In the first rehearsal there were three occurrences of a long pattern; occurring once during rehearsal of Piece 1, and twice for Piece 2.

The pattern was:

(((SOME, M SOME, M) X,I)Y, C ALL, M))

In this pattern, a subset of singers (‘SOME’) rehearsed an extract, after which Singer X Initiated further suggestions or ideas. Singer Y offered Clarification relating to what was needed, and they all sang a passage together. There was a total of 21 occurrences of two dyadic sub patterns, and the first dyadic pattern occurred within the first minute. Both dyadic interactions comprised group events (those coded ‘ALL’), so whilst they may represent the origination of a longer pattern, in this instance they do not represent specific, nascent social relationships. However, it highlights a significant role for Singer Y as there are 14 instances when an idea or clarification offered by Singer Y is followed shortly afterwards by a singing episode.

#### Group 2, rehearsal 2 (week 3)

The main pattern in Rehearsal 2 was short and had four occurrences.

The pattern was:

((X,C Y,C) ALL,M)

In the main pattern, Singers X and Y exchanged task Clarifications, followed by the whole group singing. There were 5 occurrences of the dyadic sub pattern between Singer X and Y, suggesting this as an important developing interaction. The second part of Rehearsal 2 was cut slightly short as one member had to leave the room for a few minutes.

#### Group 2, rehearsal 3 (week 6)

In Rehearsal 3 the main pattern was:

((X,I V,R)(Z.R X,I))

In the main pattern Singer X Initiated an activity, to which Singer V Reacted. This was followed by an exchange between Singers Z and Singer X – who again Initiated an idea. There were 9 occurrences of the dyadic sub patterns.

#### Group 2, rehearsal 4 (week 8)

Rehearsal 4 was more highly patterned than Rehearsal 3. The main pattern was:

((W,C Z,C)(((W, I Y,C) All, M)((W,C V,C) Y,C)))

**Figure 3:**
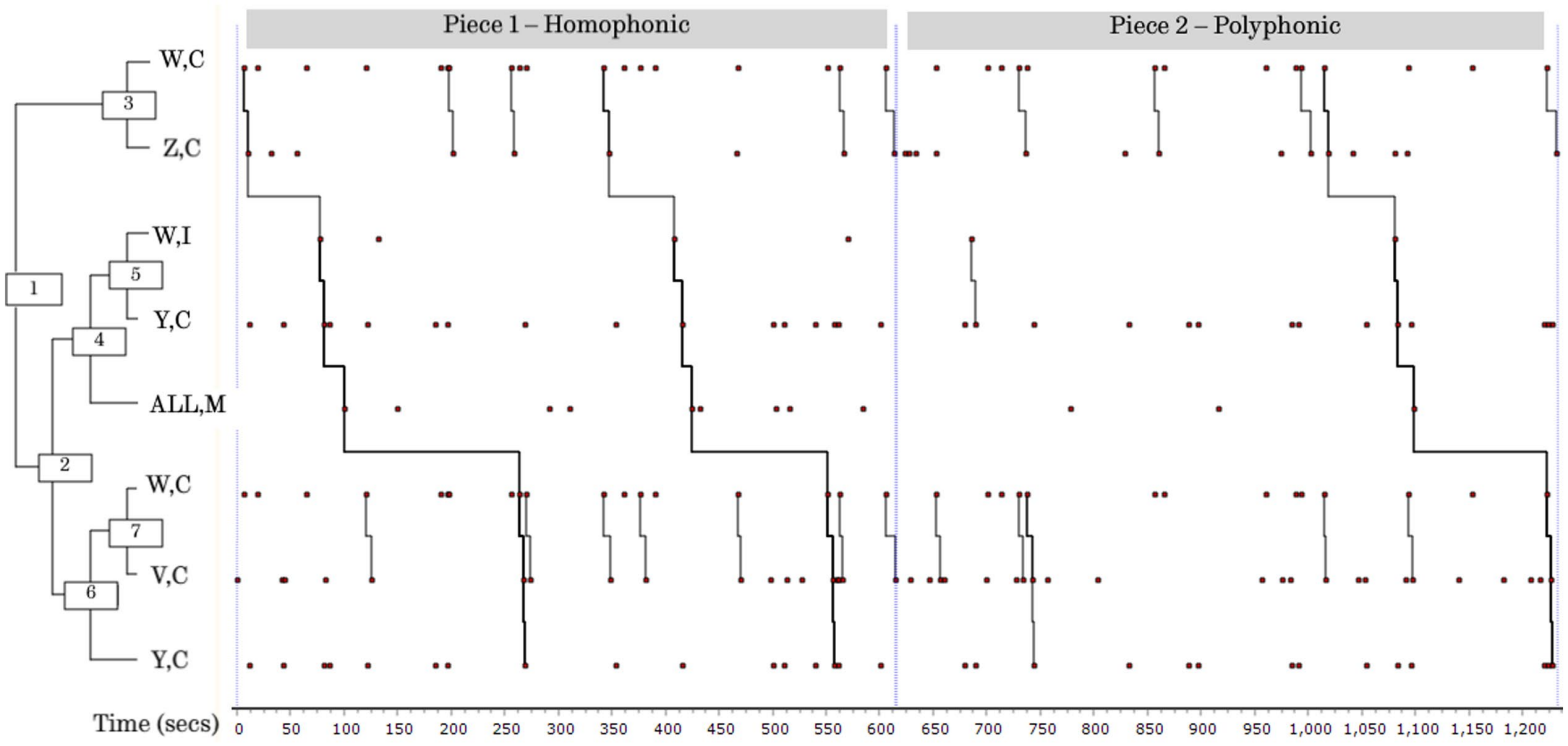
Pattern diagram (dendrogram) from THEME^®^ showing main patterns and frequent sub patterns (Group 2, Rehearsal 4). This pattern involves change of piece and shows persistence of sub patterns. The whole pattern ran as follows: first Singer W and Singer Z engaged in Clarifying behaviour (3). Singer W then Initiated, and Singer Y Clarified, after which All sang a passage (4). Singers W and V Clarified (7) and, finally, Singer Y Clarified (6). There were a large number of dyadic sub patterns (30 in total) distributed across the session, all featuring Singer W (3, 5, 7).

In the main pattern, which was dominated by Clarifying exchanges, Singer W made multiple contributions to Clarify and Initiate. Singer Y also featured prominently, both in the sub pattern prior to the singing episodes, suggesting he was providing direction or otherwise prompting the group to try an idea, and also following on from a dyadic exchange between Singers W and V. This was the most highly patterned rehearsal for Group 2. The dendrogram is shown in Figure 3, indicating the change of piece and how the sub patterns persisted across rehearsals of the two pieces.

#### Group 2, rehearsal 5 (week 16)

Rehearsal 5 was also highly patterned.

The main pattern was:

(((Y, I (Z,R ALL, M))(V,I Y,R)(Z,P (V,P Z,P)))

The main pattern in Rehearsal 5 included Participating behaviours, which were light-hearted or social in nature, and featured Singer Z more than in previous patterns. The pattern ran as follows: Singer Y Initiated an action, to which Singer Z Reacted, followed by All singing. Singer V Initiated, and Singer Y Reacted. Finally, Singer Z engaged in Participating behaviour, then Singers V and Z exchanged Participating behaviours. There was a total of 18 dyadic sub patterns.

In summary, the following informal roles were identified:

Singer V: In a prominent vocal role when singing but only featured in patterns from week 3.

Singer W: Quiet at first, increasingly active in final two rehearsals.

Singer X: Often initiated, contributed and reacted in first three session.

Singer Y: Active in all rehearsals, often initiating and reacting.

Singer Z: Generally quiet, active in rehearsal 3, but most contributions in rehearsal 5.

Patterns in Weeks 1 and 3 only included Singers X and Y, but in later rehearsals more singers contributed. Notably, Singers W and Z were initially quiet, but became more active in weeks 8 (W) and 16 (Z).

#### Changing task requirements of the musical material in group 2

The number of patterns, events, actor switches and dyads occurring during the rehearsals of Pieces 1 and 2, are shown in Table 6. There were three main patterns detected in all except Rehearsal 4, where there were 4. The number of events was highest in Rehearsals 4 and 5. The number of dyadic interactions in rehearsals ranged from 5 (Rehearsal 2) to 30 (Rehearsal 4). In all except Rehearsal 2, more dyadic patterns are evident during rehearsals of Piece 1 (mean = 9.2, S.D. = 5.8) than Piece 2 (mean = 7.4, S.D. = 4.2). Actor switches were most active in the final two rehearsals (Rehearsals 4 and 5), which is consistent with the observation of more members of the group contributing to the discussion.

**Table 6:** Summary of main patterns by rehearsal and piece; number of main patterns, number of dyads, number of actors, duration and actor switches, and talk time as % of session time.

Rehearsal	Piece order	Number of patterns	Number of dyads	Duration (secs) (mean)	No of actors (mean)	Actor switches (mean)	Levels (mean)	Amount of talk (% session)
1	H	1	11	2.17	2.00	1.00	1.17	59.5
	P	2	10	2.40	2.13	1.27	1.33	70.2
		3	21	2.29	2.07	1.14	1.25	64.9
2	P	3	3	2.20	2.00	1.00	1.20	61.4
	H	1	2	2.00	2.00	1.00	1.00	53.3
		4	5	2.10	2.00	1.00	1.10	57.4
3	P	2	4	2.11	1.78	1.11	1.11	65.1
	H	1	5	2.36	2.05	1.36	1.36	71.9
		3	9	2.24	1.92	1.24	1.24	68.5
4	H	2	17	2.58	2.33	1.33	1.58	73.0
	P	1	13	2.33	2.33	1.33	1.33	84.4
		3	30	2.46	2.33	1.33	1.46	78.7
5	H	2	11	2.15	1.77	0.77	1.15	51.4
	P	1	7	2.94	2.34	1.66	1.72	59.1
		3	18	2.55	2.06	1.22	1.44	55.3

The interaction pattern data shows a change in pattern event and dyad frequency after Rehearsal 3, and considerable variation across rehearsals in contributions and behaviour types. There were no marked differences between piece types. Although Rehearsal 1 did show a largish number of dyads, in other respects it was comparable to Rehearsals 2 and 3 in terms of number of actor switches, mean duration of pattern, and length. Indeed, up to Rehearsal 3, the number of events making up a pattern was steady, the amount of turn-taking was low (as indicated by actor switches) and the mean duration was relatively low. Rehearsals 4 and 5 showed an increase in all three measures (Table 5), with the longest patterns and most turn-taking in Rehearsal 5. There was a change between Rehearsals 3 and 4, with an increase in pattern complexity, which persisted to Rehearsal 5. The increase in pattern events over time suggests that sequences of individual contributions were sustained for longer. It may be that the group were experimenting with different ways of interacting up to Rehearsal 3.

From the pattern descriptions it is apparent that qualitatively they differ from each other too – Rehearsal 4 has more of a Clarifying task emphasis, consistent with a focussed, problem-solving approach, whilst Rehearsal 5 patterns are more light-hearted in tone, including more Reacting, Initiating and Participating behaviour. Notably, these two rehearsals also incorporate episodes of ‘All singing’ as part of the main patterns, reinforcing their focus on performance outcomes. The number of dyadic sub patterns is greatest in Rehearsal 4. This supports the prediction of the team compilation model advanced by Kozlowski et al. (1999), in which dyadic interactions increase over time but are ultimately a stage towards holistic team function.

**Figure 4:**
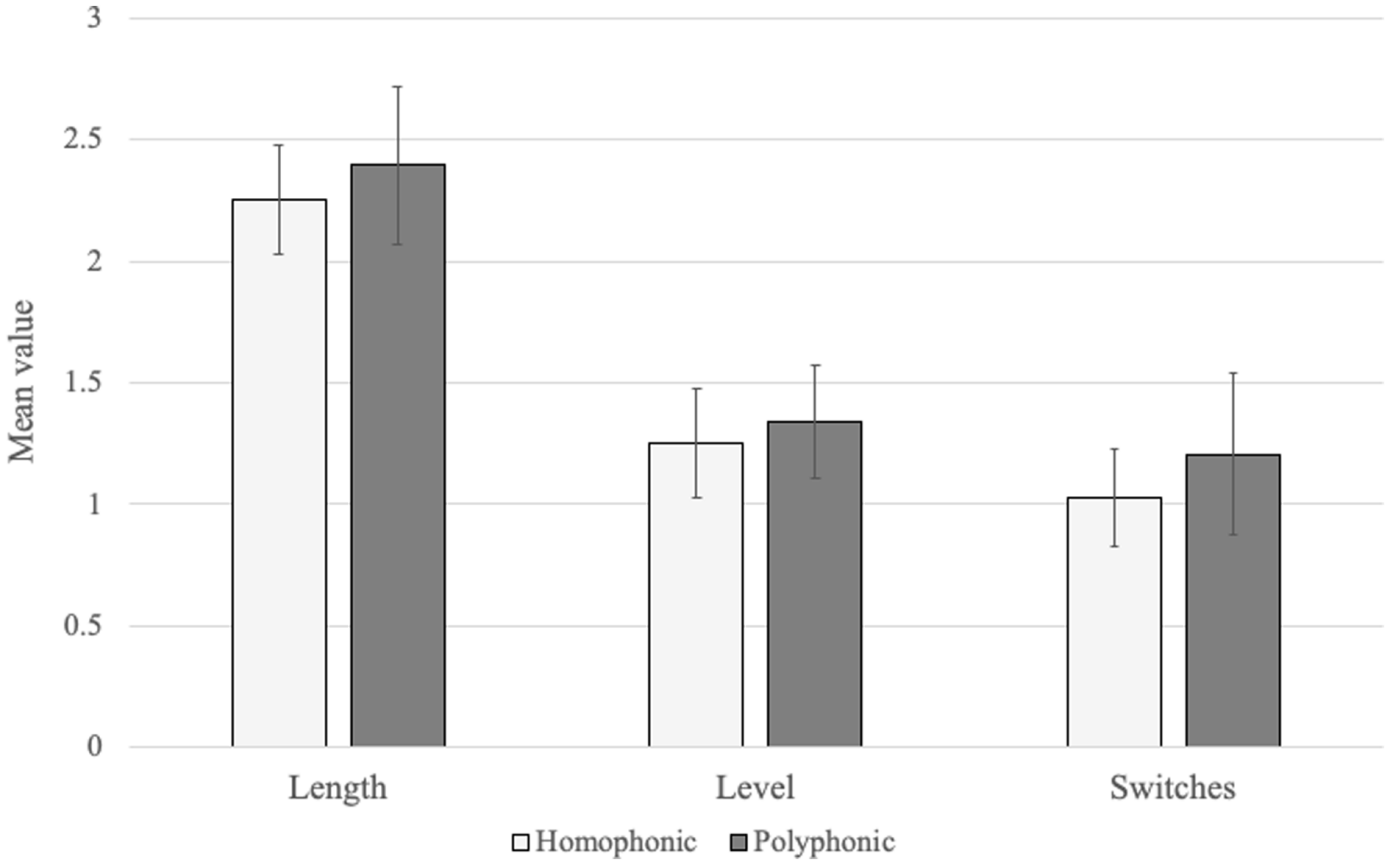
Mean value and standard deviation of pattern length, number of levels, and number of actor switches, by homophonic and polyphonic piece structure (Group 2).

Pattern length (duration), level (number of levels in the hierarchy of patterns) and number of actor switches were summarised by piece structure (Table 6; Figure 4). For the polyphonic piece, patterns were observed to be generally longer, more complex and with more actor switches, so may warrant further investigation with larger samples. These observations together suggest that the more ‘complex’ musical task (in this case rehearsal of the polyphonic piece) is associated with more patterned behaviour and greater amount of talk. However, other measures were less conclusive, as dyadic interactions and number of patterns tended to be greater during the homophonic piece.

The distribution of main patterns between the segments of rehearsal allocated to the first or second piece that was rehearsed was not consistent. In Rehearsals 1–3, more patterns appeared in the second rehearsed piece segments; however, in Rehearsals 4 and 5 this was reversed. It may rather be an order effect – in all except Rehearsal 1 the first segment contained more patterns, regardless of whether the piece was homophonic or polyphonic. Again, sample sizes were not large enough to examine this statistically.

In summary, there were some indications from these findings that piece structure may influence behaviour and perceptions of group interactions, resulting in more talk and more complex interactions during rehearsal of more complex material. Future research with a larger sample and even more contrasting musical material could usefully explore whether rehearsals of pieces of different structure or complexity indeed result in differences in amount and type of verbal interactions, and in complexity of patterns.

## Discussion

In both groups, there were observed changes over time in the amount of talking and singing and developing patterned interactions. The observed changes suggest transitional shifts in the group’s collaborative processes as evidenced in verbal interactions and patterns of behaviour. We consider in our discussion the role of the individual in the group, implications for underlying structure and transitions for group development, and how these findings may contribute to the ongoing study of small groups as complex adaptive systems.

### Changes in interaction patterns

Early patterns formed in both groups and were evident even in very short rehearsals investigated in Group 2. In Group 1, patterns appeared within 2 min, in Group 2 within 1 min. There was an increase in pattern number and complexity over time, even though less time was spent talking. More group members were involved in patterns in later sessions. Appearance of dyadic patterns, most marked in Group 1, but also present in Group 2, may provide a mechanism to support the formation of longer patterns (Kozlowski et al., 1999). In both groups, roles and contributions in the patterns were flexible and involved all group members in different formations, depending on rehearsal. This ongoing emergence and changing of interaction patterns, happening over short time periods (seconds and minutes, as well as the larger timescale of a series of rehearsals) reflects moments of incremental as well as longer-term changes over time, showing fluidity rather than rigidity in interaction.

The larger scale changes evident in Group 1 of an increase followed by a decrease in pattern complexity was only partially apparent in Group 2, who showed an initial, limited increase in complexity. A difference here was that Group 1 had a longer-term goal to which all members were fully committed and engaged with (a final, assessed performance), whilst Group 2 were set goals by the researchers which were appropriate for the laboratory setting. Alongside this, however, Group 2 were working on their own material for a planned assessment, so some evidence of group development was to be expected. Even though the two musical tasks given to Group 2 had different levels of demand, there may have been a sense in which from the second rehearsal both became more ‘routine’, as the group knew what to expect, whereas Group 1 were operating in what could be described as a more knowledge-intensive, non-routine environment as they were consistently exposed to new experiences and material. In such a context, there is a demand for more sharing and interpretation of complex information amongst team members (Kozlowski and Bell, 2003), with greater need for exchange of ideas and development of a shared understanding of the changing task environment (Lei et al., 2016). Looking at complexity of tasks in teams, Hoogeboom and Wilderom (2019) found a stronger effect of participative team interaction patterns in a nonroutine (versus routine) task context. Hence, the environment in which Group 1 was operating may have allowed more inclusive patterned behaviour to develop and adapt to the changing task demands week by week. On the other hand, working on routinised tasks as was more characteristic for Group 2 can offer opportunities for sharing of expertise, social bonding, and planning (Marks et al., 2001; Chung and Jackson, 2013). Indeed, the final rehearsal of Group 2 was largely social featuring behaviours of Participation and Reaction rather Clarifying.

No major differences were found in pattern type and complexity with change of piece (Group 2), suggesting that the level of shared knowledge was sufficient to provide resilience to a change of task (Uitdewilligen et al., 2018). We recognise the limitations of the sample size and the analysis method as analyses were conducted across the two pieces, taking the rehearsal as a whole. Exploring the extent to which changing rehearsal material (a very usual practice for musicians) represents changing task demand remains an area of potential further study.

### The role of the individual

The simpler patterns found in both groups in Week 1 illustrate how the groups were getting started, where individual members may be drawing on their existing knowledge of normal, ‘routine’ rehearsal practises without getting into elaborate discussions. As the weeks progressed, the roles of group members changed, evidenced by their degree of involvement in interaction patterns, and in emerging specialisms within the group, for example, the tendency of Singer D to contribute light-hearted comments that triggered a change in activity. In Group 2, Singers W and Y tended to contribute more task-focussed (‘Clarifying’) behaviours, particularly expressed in Rehearsal 4. These examples illustrate how individual roles not only serve to influence the interactions in the group, but also how the patterns identified by THEME^®^ can provide further insights into the roles of individuals and their development.

As they became familiar with each other and rehearsing together, the groups had more time to explicitly coordinate their work and to anticipate the actions of others. The presence of mono-actor patterns may indicate a lack of balance in contributions from all team members, resulting in lower effectiveness of group working (Zijlstra et al., 2012). We found that mono-actor patterns were relatively infrequent, suggesting that a good balance of contributions was achieved. For Group 1, there were three mono-actor patterns in Week 5; whilst there are other differences in the patterns in Week 5, this may reflect the absence of one group member. Dyadic sub patterns were a feature of Weeks 3 and 5 (Week 3: Singers B and E, 27 occurrences; Week 5: Singers C and E, 19 occurrences) which may be an indicator of the development of social relationships. A ‘contagion’ effect was reported in basketball teams by Bourbousson et al. (2015), whereby the presence of tightly coupled dyads made it easier for a third member to join and create a triad, resulting in longer patterned interactions. In Group 2, the number of dyadic sub patterns was greatest in Rehearsal 4, reducing in Rehearsal 5, suggesting integration beyond dyadic relationships.

### Structure and transitions

One way in which these changes can be interpreted is as a series of emergent phases. First, the early encounters provided opportunities for the groups to self-organise and establish patterns of behaviour, and social relationships. The groups sought to gain knowledge of one another to establish order, to be able to predict the behaviour of their fellow group members (Okhuysen and Bechky, 2009) and establish a flow to their interactions (van Oortmerssen et al., 2015). These early interaction patterns enable and facilitate progress in unfamiliar teams, by providing a mechanism to quickly establish a balanced communication involving multiple (although not necessarily all) members (Zijlstra et al., 2012). The patterns are generally ‘hidden’ from the group members, and obscured by overt, vocal exchanges, especially by dominant personality types. In Group 1, early patterns involved a shared task and three group members. It is also notable that in Week 1 the most vocal member (Singer B) does not feature in the pattern, reinforcing the idea that the patterned behaviours exist at a different level of interaction as Singer B did contribute strongly to the first rehearsal in terms of total speaking time (Pennill, 2019). The non-conscious and unfolding patterns of interaction may therefore enable ‘quieter’ members to contribute and for their influence to be expressed and endure through patterns in small group contexts. After the initial emergence of simple, short patterns, further developments were apparent. This was apparent in an increase in length and complexity of the interactions and the number of people involved. In Group 1, there was a marked change in patterned behaviour in Week 5, as patterns increased in length, and with more people and switching involved in the discussions. This also coincided with the calendar midpoint, as deadline pressure starts to become more urgent (Gersick, 1988, 1989). In Group 2, a tipping point was less evident, although there was an increase in pattern length and duration in Rehearsal 4 (the calendar midpoint), this increase persisted to Rehearsal 5. For the final stage of Group 1, there were signs of integration, or convergence.

To interpret these developments, it is helpful to draw on the framework by Okhuysen and Bechky (2009) in which the three pillars of accountability, predictability and common understanding contribute to a shared sense of alignment, integration, and, ultimately, coordination. In dancers, divergence and even separation (‘de-integration’) have been shown to be important precursors to group coordination (Harrison and Rouse, 2014). In Group 1, the simple, short patterns are consistent with integration (a coming together of ideas) in Weeks 1 and 3. However, the increase in length and complexity suggests a period of de-integration in Week 5, followed by further integration of ideas and interactions as patterns simplify in Week 7. In Group 1, there was a marked convergence in Week 7 as patterns showed their strongest self-similarities – not only between group members, their timing and type of behaviour, but also in the content of what was addressed. The high degree of similarity in both interactions and musical content suggests an effect of increasing familiarity and the influence of developing predictability of contribution. As with other types of work groups, achievement of integration proceeds in a cyclical or episodic, rather than linear way (Marks et al., 2001). An episodic process of integration is also evident in Group 2. Over the five rehearsals, the patterns show increasing complexity to Rehearsal 4, as measured by the number of hierarchical levels and constituent events. Given that there was an aim for coherence and convergence in the output of the group, Rehearsal 4 represents a pivotal session in creating conditions for further integration in Rehearsal 5. In terms of elapsed time, Rehearsal 4 occurs at the mid-point of the timeline. In later rehearsals, there was an emerging sense of integration which was evident in both groups. In Group 2, more group members (as measured by ‘actor switches’) were involved in the patterns over time. This also reflected the willingness amongst members for more involvement and created more balanced team interactions, contributing to greater common understanding and accountability.

### Adaptation and emergence

In this research we found evidence of adaptation and emergence, which are features of complex adaptive systems (CAS). In their research on adaptation in teams, Kozlowski et al. (1999) suggest that dyad formation is an indicator that a team is forming social bonds and developing task mastery in response to changing stimuli. It is also consistent with the theory of small groups as complex systems, which relates the achievement of coordination goals to “ongoing patterns of interaction amongst the group’s constituent elements as the group pursues its function” (Arrow et al., 2000, p. 55). A further example of this arose in Group 2, where complex patterning was retained over a break. This may be an effect of attunement to the task, whereby patterns that fit the task requirement tend to be retained (Uitdewilligen et al., 2018). Interaction patterns generally increased in complexity over time. This is consistent with research in other dynamic work situations, where teams demonstrated increasing pattern complexity (Lei et al., 2016; Uitdewilligen et al., 2018; Hoogeboom and Wilderom, 2019). This emergent behaviour may have been impacted by the setting; whilst the groups were rehearsing independently, they were working within the framework provided by a higher education institution. Any coaching or guidance offered was outside the scope of the study but may have had an impact on the ways that the students approached their rehearsals. Indeed, emergent self-expression in dance performance students was found to be moderated by the way tasks were framed (Torrents et al., 2013). There may be other, more subtle, mechanisms at work, too, in the way the groups moved towards more balanced and inclusive interactions, however, there were many parallels with the ways that professional musicians have been shown to prepare for performance (Ginsborg, 2017).

### Concluding remarks

This research took an organisational approach to better understand how musicians work together on tasks which are related to their real-world experiences. It demonstrated the feasibility of investigating communication in ensembles longitudinally using BA and T-pattern analysis and the powerful, richness of insight. The contribution of individual group members, the types of verbal behaviours exhibited, and the patterns identified within them contributed to our understanding of how these newly formed groups established aspects of their musical practice.

Both groups came together as experienced musicians who had diverse knowledge but shared a common understanding of the conventions of vocal ensemble rehearsal in their chosen genre. This shared vocabulary and experience enabled them to quickly establish effective ways of working. Their homogeneity in stage of career, prior training and chosen musical genre contributed to a solid basis for collaborative work and progression. Within this broad structure, the roles of group members emerged and changed, evidenced by their degree of involvement in interaction patterns, and in their vocal specialisms within the group.

A focus on verbal behaviours, their frequency and patterns highlight qualitative differences too. For example, Clarifying behaviours were a feature of the longer, more complex patterns, and related to problem-solving activities or information gathering by group members as discussions or explorations unfolded. Conversely, Reacting behaviours appeared in the simpler patterns. In several instances, Participating behaviours were a trigger for shift in focus or activity. The patterns within these interactions provided a way to explore the ‘flow’ of the groups over time, and the ways that simple and complex patterns emerged at different points, for example in Group 1, an increase in pattern complexity as the challenges of the material and deadline pressure increased.

This study contributes to an ongoing exploration of human group behaviour and interaction patterns in team performance. In the specialised context of European classical music, musicians rehearsing for a future performance, there is an intentional shift to nonverbal (more implicit) processes (King and Gritten, 2017). Observed changes in behaviour and interaction patterns, both over time and with changing tasks, are consistent with emergence of these implicit behaviours (Rico et al., 2008).

## Data availability statement

The datasets presented in this study can be found in online repositories. The names of the repository/repositories and accession number(s) can be found at: The University of Sheffield’s online data repository (ORDA) 10.15131/shef.data.20231877.

## Ethics statement

The research was reviewed and approved by the Physical Science Ethics Committee (PSEC) at the University of York (United Kingdom), reference 070817. The participants provided their written informed consent to participate in the study.

## Author contributions

NP was the principal investigator and made substantial contributions to the conception and design of the study, data acquisition, analysis, and interpretation. She drafted the article and approved the submitted version. RT acted as supervisor of the research, contributed to the design of the study and interpretation of the results, critically revised the article and approved the submitted version.

## Funding

This research was funded by an Arts and Humanities Research Council (AHRC) WRoCAH studentship. Open access publication fees were supported by a UKRI grant to The University of Sheffield.

## Conflict of interest

The authors declare that the research was conducted in the absence of any commercial or financial relationships that could be construed as a potential conflict of interest.

## Publisher’s note

All claims expressed in this article are solely those of the authors and do not necessarily represent those of their affiliated organizations, or those of the publisher, the editors and the reviewers. Any product that may be evaluated in this article, or claim that may be made by its manufacturer, is not guaranteed or endorsed by the publisher.

## Footnotes

^1^ Magnusson, (2000).
